# ESCRT-III controls nuclear envelope deformation induced by progerin

**DOI:** 10.1038/s41598-020-75852-6

**Published:** 2020-11-02

**Authors:** Jun Arii, Fumio Maeda, Yuhei Maruzuru, Naoto Koyanagi, Akihisa Kato, Yasuko Mori, Yasushi Kawaguchi

**Affiliations:** 1 Division of Molecular Virology, Department of Microbiology and Immunology, The Institute of Medical Science, The University of Tokyo, 4-6-1 Shirokanedai, Minato-ku, Tokyo, 108-8639 Japan; 2 Department of Infectious Disease Control, International Research Center for Infectious Diseases, The Institute of Medical Science, The University of Tokyo, Tokyo, Japan; 3 Research Center for Asian Infectious Diseases, The Institute of Medical Science, The University of Tokyo, Tokyo, Japan; 4 Division of Clinical Virology, Center for Infectious Diseases, Kobe University Graduate School of Medicine, Kobe, Hyogo Japan

**Keywords:** Cell biology, Molecular biology

## Abstract

Hutchinson-Gilford progeria syndrome (HGPS) is a premature aging disorder, caused by mutation in the gene encoding lamin A/C, which produces a truncated protein called progerin. In cells from HGPS patients, progerin accumulates at the nuclear membrane (NM), where it causes NM deformations. In this study, we investigated whether progerin-induced NM deformation involved ESCRT-III, a protein complex that remodels nuclear and cytoplasmic membranes. The ESCRT-III protein CHMP4B was recruited to sites of aberrant NM proliferation in human cells ectopically expressing progerin and in patient-derived HGPS fibroblasts. Derepression of NM deformation in these cells was observed following depletion of CHMP4B or an ESCRT-III adaptor, ALIX. Treatment with rapamycin (which induce autophagic clearance of progerin and reverse progerin-induced cellular phenotypes) down-regulated progerin-induced NM deformation, whereas treatment with bafilomycin A1 (an inhibitor of autophagy and lysosome-based degradation) or CHMP4B depletion antagonized the effects of rapamycin. These results indicate that the ALIX-mediated ESCRT-III pathway plays a suppressive role in progerin-induced NM deformation and suggest that autophagy down-regulates progerin-induced NM deformation in a manner dependent on ESCRT-III machinery.

## Introduction

By exerting cellular membrane deformation and scission, endosomal sorting complex required for transport-III (ESCRT-III) mediates multiple cytoplasmic processes including intraluminal vesicle formation, extracellular microvesicle formation, enveloped virus budding, and the abscission stage of cytokinesis^1^. In addition to these well-characterized cytoplasmic functions of ESCRT-III, the complex has nuclear roles including resealing NMs during late anaphase^2,3^, quality control of nuclear pore complex (NPC) assembly^4,5^ and repair of NM ruptures that are produced during the migration of cancer and immune cells^6,7^. We recently reported that ESCRT-III is required to maintain the integrity of the inner nuclear membrane (INM) in human cells and to cleave the INM to produce vesicles containing macromolecular complexes such as *Drosophila* large ribonucleoprotein complexes (RNPs) and progeny nucleocapsids of herpes simplex virus 1 (HSV-1) during their vesicle-mediated nucleocytoplasmic transport^8^.

Hutchinson-Gilford progeria syndrome (HGPS) is a premature aging disorder, with an early onset, fast progression and a 100% mortality rate^9,10^. HGPS patients have an aged appearance, and in the final stages of the disease, most children have hypertension, angina, and dilated hearts because of atherosclerotic heart disease^9,10^. HGPS is primarily caused by a silent point mutation in the lamin A/C gene^11,12^, which creates an abnormal splice donor site producing a truncated protein (progerin) lacking 50 amino acid residues near the C terminus. In primary patient-derived HGPS fibroblasts, progerin accumulates in the nucleus in a cell age-dependent manner, inducing intranuclear aggregates of progerin, nuclear blebbing caused by NM deformations, a reduced growth rate and broad epigenetic alterations in histone methylation^13–16^. However, the intrinsic mechanisms for regulation of progerin-induced NM deformation and progerin accumulation and clearance are poorly understood. Notably, induction of autophagy by drugs such as rapamycin and MG132 leads to progerin degradation in HGPS fibroblasts, thereby reversing progerin-induced cellular phenotypes^17,18^. These observations suggest that autophagy is involved in regulation of progerin turnover in the nucleus^17,18^. However, autophagosomes are present only in the cytoplasm, raising the intriguing question of how progerin at the NM is transported to the cytoplasm.

During the course of our earlier study showing the role of ESCRT-III in the maintenance of INM integrity^8^, we noticed that NM deformation caused by depletion of a critical component of ESCRT-III in human cells was similar to progerin-induced NM deformation in HGPS fibroblasts^15,16^. Therefore, in this study, we investigated the role of ESCR-III in progerin-induced NM deformation.

## Results

### ESCRT-III is recruited to the NM in cells ectopically expressing progerin

**Figure 1:**
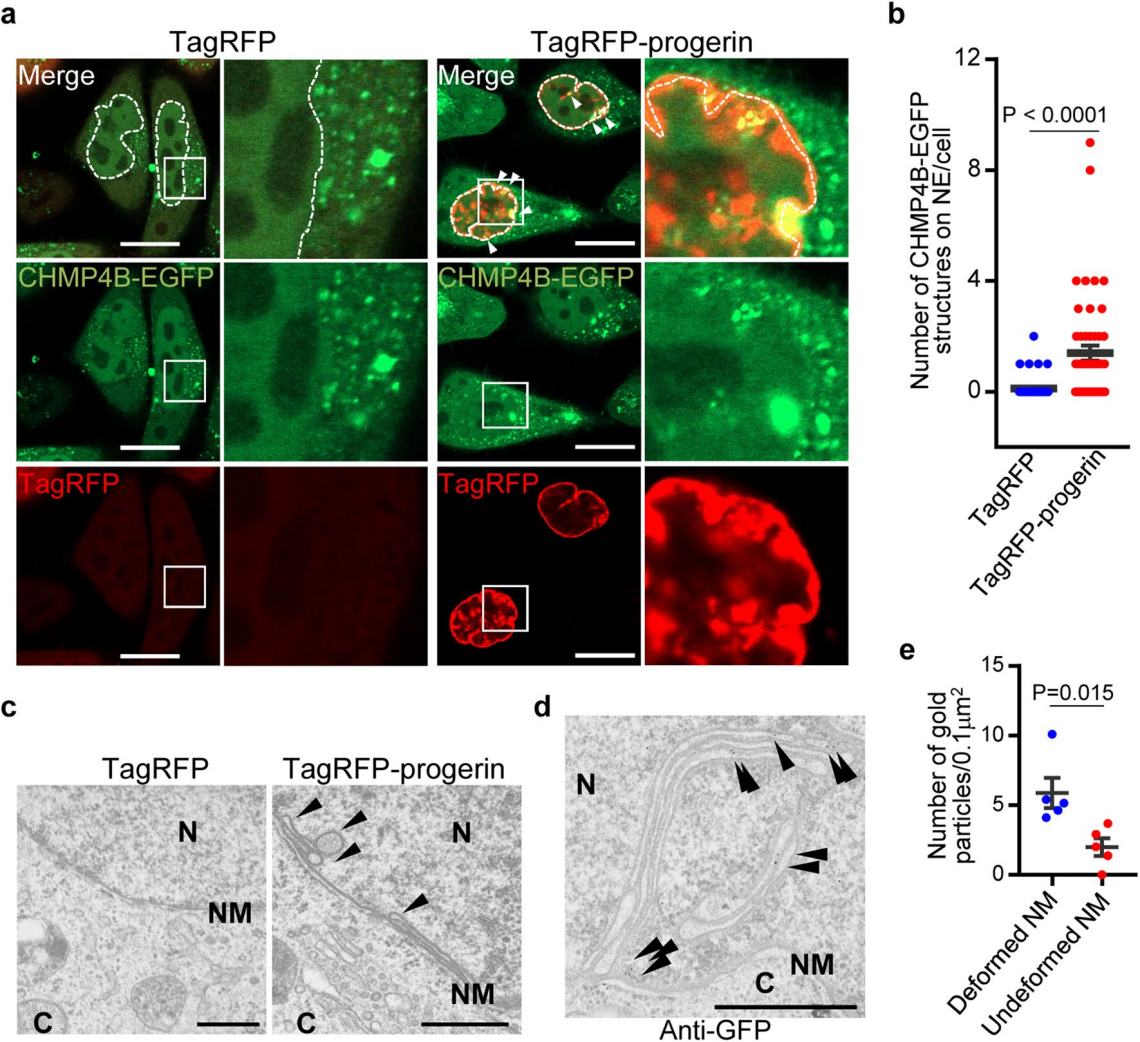
ESCRT-III is recruited to sites of membrane proliferation induced by the ectopic expression of TagRFP-progerin. (**a**) Confocal images of HeLa/CHMP4B-EGFP cells transfected with the TagRFP or TagRFP-progerin expression vector for 24 h. The right panel is the magnified image of the boxed area in the left panel. Dotted lines indicate the NMs in TagRFP-transfected cells. Arrowheads indicate punctate structures with CHMP4B-EGFP at the nuclear rim. Bars, 20 µm. Images are representative of 3 independent experiments. (**b**) The number of CHMP4B-EGFP nuclear punctate structures was measured in 50 transfected HeLa cells in the experiment in (**a**). Data are shown as the mean ± SEM and are representative of 3 independent experiments. (**c**) Electron microscope images of cells transfected with TagRFP-progerin or the TagRFP expression plasmid for 24 h. Arrowheads indicate transnuclear tubes derived from the INM. N, nucleus; C, cytoplasm; NM, nuclear membrane. Bars, 500 nm. Images are representative of 3 independent experiments. (**d**) Immunoelectron microscope images of HeLa/CHMP4B-EGFP cells transfected with the TagRFP-progerin expression vector for 24 h. Arrowheads indicate localization of CHMP4B-EGFP at the invagination structures detected by anti-GFP antibody. Bars, 500 nm. Images are representative of 3 independent experiments. (**e**) Quantitation of gold particles on the NM in the experiment in (**d**). Five areas of each of the sections were analyzed and data are shown as the mean ± SEM (n = 5) and are representative of 2 independent experiments.

In HGPS fibroblasts, both wild type lamin A/C and the truncated protein, progerin, are expressed. Consistent with this, ectopic expression of progerin in normal cells induces NM deformation as observed in HGPS fibroblasts^15,16^. To investigate the role of ESCRT-III in the regulation of progerin-induced NM deformation, we first analyzed the localization of an ESCRT-III protein, charged multivesicular body protein (CHMP) 4B, in cells overexpressing progerin fused to a fluorescent protein TagRFP (TagRFP-progerin). CHMP4 proteins (CHMP4A, -B and -C) are components of the ESCRT-III complex with critical functions in membrane remodeling, with CHMP4B playing a major role^19–21^. As shown in Fig. 1a, confocal microscopic analysis showed that the ectopic expression of TagRFP-progerin in HeLa cells stably expressing CHMP4B fused to enhanced green fluorescence (EGFP) (HeLa/CHMP4B-EGFP)^8^ induced tube-like structures of TagRFP-progerin at the nuclear rim. In addition, electron microscopic analysis demonstrated that the ectopic expression of TagRFP-progerin in these cells induced proliferation of the INM (Fig. 1c). These results are in agreement with previous reports^15,16^. Notably, the INM proliferation in Fig. 1c,d was reminiscent of the karmellae layers and whorls of NM tubules that are detected in yeast lacking each of the INM and ESCRT-III proteins^22–24^. This suggests that TagRFP-progerin induces aberrations in the 3D structure of the NM, which in turn leads to NM deformation.

In particular, punctate structures with CHMP4B-EGFP detected by confocal microscopy were specifically induced at the nuclear rim and co-localized with TagRFP-progerin tube-like structures (Fig. 1b). In addition, CHMP4B-EGFP was detected by immunoelectron microscopy in the proliferated INMs of these cells (Fig. 1d). Immunoelectron microscopy showed that the CHMP4B-EGFP accumulation was more frequent in the deformed INM than in the undeformed INM (Fig. 1e); this is in agreement with the results above where CHMP4B accumulated at the tube-like structures of TagRFP-progerin detected by confocal microcopy (Fig. 1a,b). These results indicate that CHMP4B is recruited to the sites of NM proliferation induced by progerin.

### ESCRT-III down-regulates progerin-induced INM deformation

**Figure 2:**
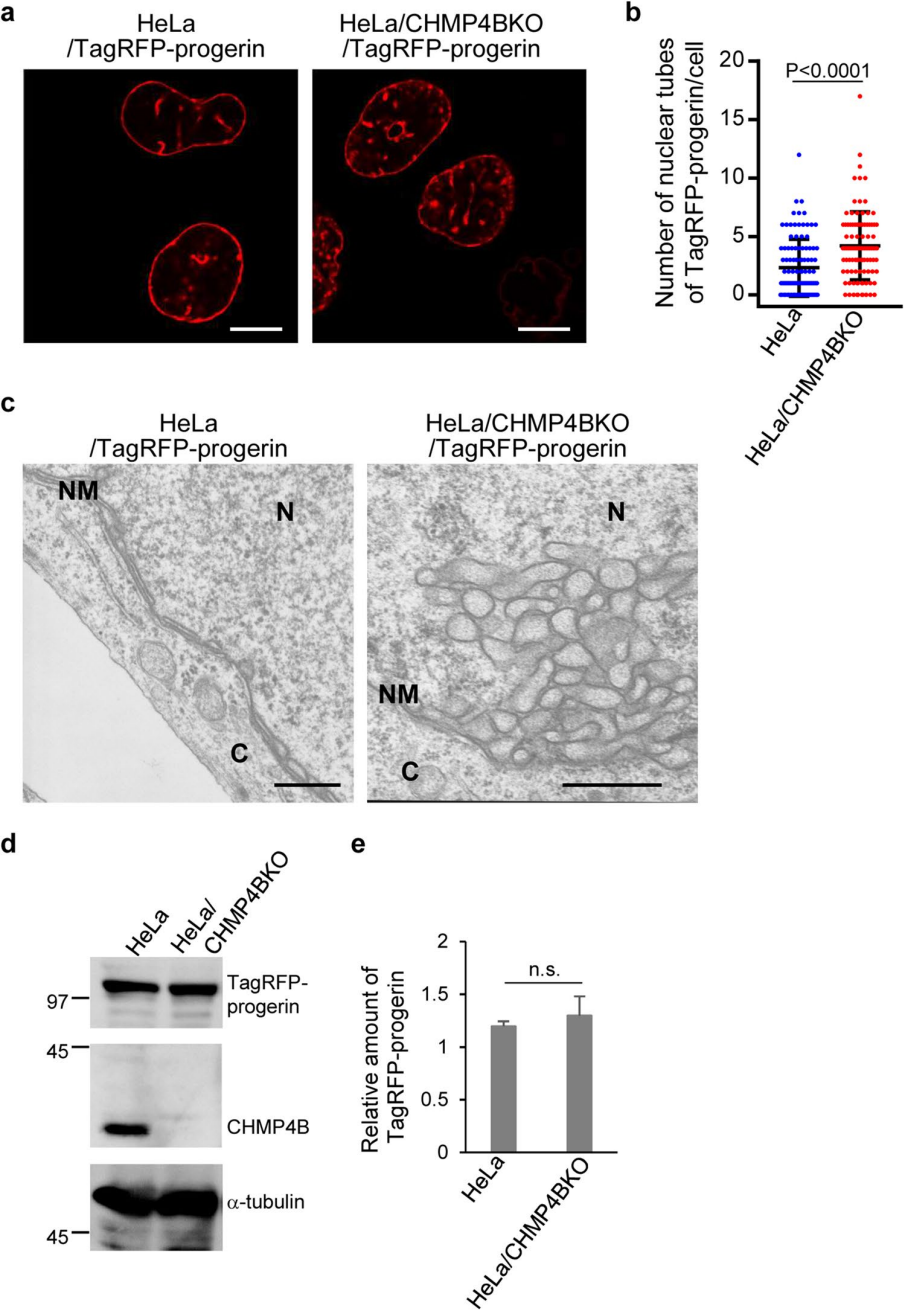
Membrane proliferation induced by the ectopic expression of TagRFP-progerin is de-repressed by ESCRT-III. (**a**) HeLa cells were transfected with the TagRFP-progerin expression vector for 24 h and analyzed by confocal microscopy. Bars, 20 µm. Images are representative of 3 independent experiments. (**b**) The number of nuclear tube-like structures was measured in 105 transfected cells in the experiment in (**a**). Data are shown as the mean ± SEM and are representative of 3 independent experiments. (**c**) Electron microscope images of HeLa and HeLa/CHMP4BKO cells transfected with the TagRFP-progerin expression vector for 24 h. N, nucleus; C, cytoplasm; NM, nuclear membrane. Bars, 500 nm. Images are representative of 3 independent experiments. (**d**) Lysates of HeLa and HeLa/CHMP4BKO cells transfected with the TagRFP-progerin expression vector for 24 h were analyzed by immunoblotting with anti-TagRFP, anti-CHMP4B and anti-α-tubulin antibodies. Images are representative of 3 independent experiments. Full-length blots are presented in Supplementary Fig. S1. (**e**) Quantitation of the amount of TagRFP-progerin, as indicated, in the immunoblot bands shown in panel (**d**) relative to the amount in the α-tubulin band. Each value is the mean ± standard error of 3 independent experiments. *n.s.* not significant (by Student’s *t*-test).

**Figure 3:**
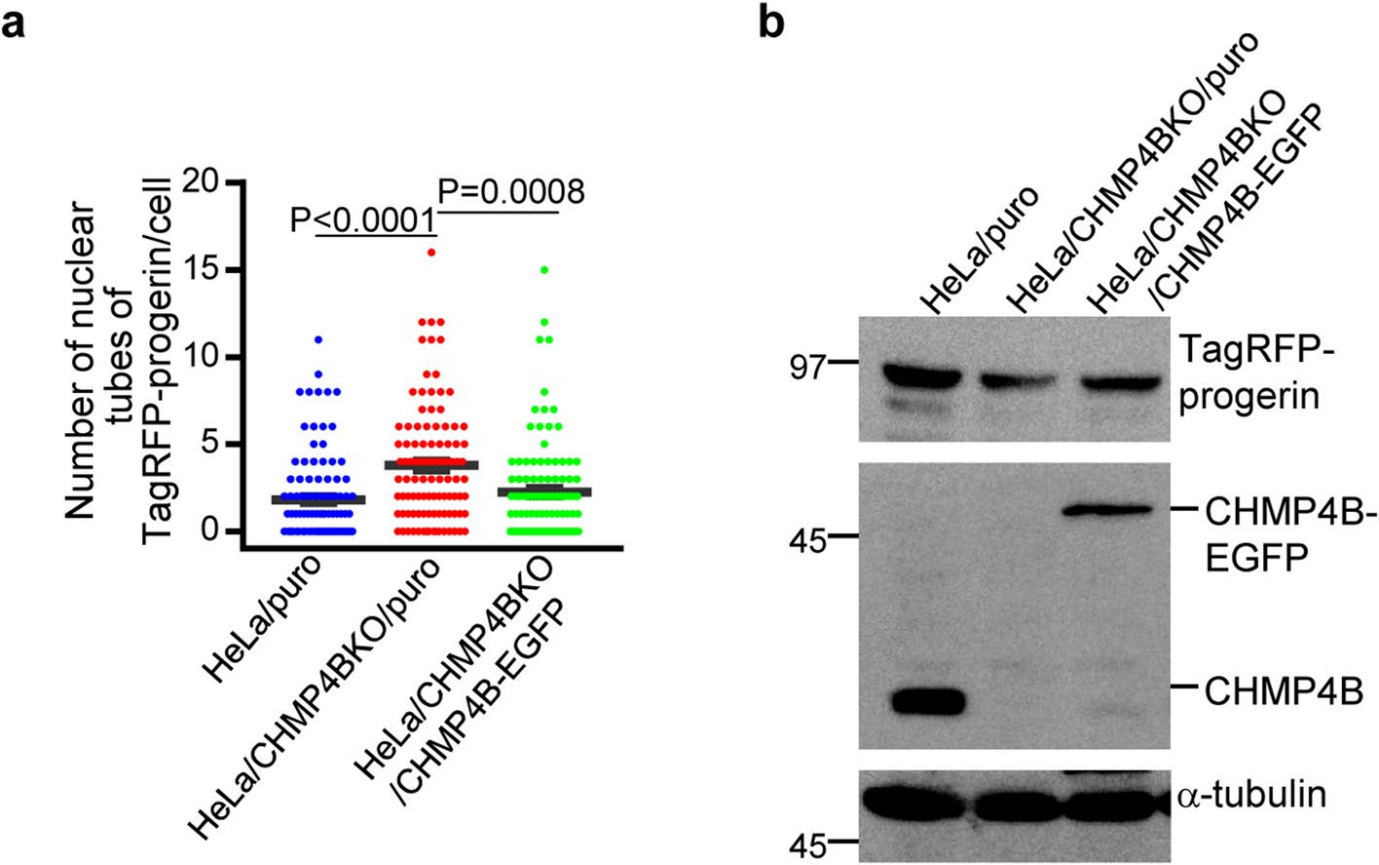
Effects of ectopic expression of CHMP4B in HeLa cells. (**a**) HeLa/puro, HeLa/CHMP4BKO/puro and HeLa/CHMP4BKO/CHMP4B-EGFP cells were transfected with the TagRFP-progerin expression vector for 24 h. The number of nuclear tube-like structures was measured in 100 transfected cells. Data are shown as the mean ± SEM and are representative of 3 independent experiments. (**b**) Lysates of HeLa/puro, HeLa/CHMP4BKO/puro and HeLa/CHMP4BKO/CHMP4B-EGFP cells transfected with the TagRFP-progerin expression vector for 24 h were analyzed by immunoblotting with anti-TagRFP, anti-CHMP4B and anti-α-tubulin antibodies. Images are representative of 3 independent experiments. Full-length blots are presented in Supplementary Fig. S2.

We next investigated the effect of ESCRT-III on the aberrant INM proliferation induced by ectopic expression of TagRFP-progerin using HeLa/CHMP4BKO cells^8^ in which CHMP4B was knocked out by the CRISPR/Cas9 system. As shown in Fig. 2, CHMP4B depletion significantly increased the number of TagRFP-progerin tube-like structures (Fig. 2a,b) and the levels of the INM proliferation (Fig. 2c) without affecting accumulation of TagRFP-progerin (Fig. 2d,e). The effect of CHMP4B depletion in these cells was significantly rescued by the ectopic expression of CHM4B-EGFP (Fig. 3a,b). These results indicate that ESCRT-III is required to suppress progerin-induced INM deformation.

**Figure 4:**
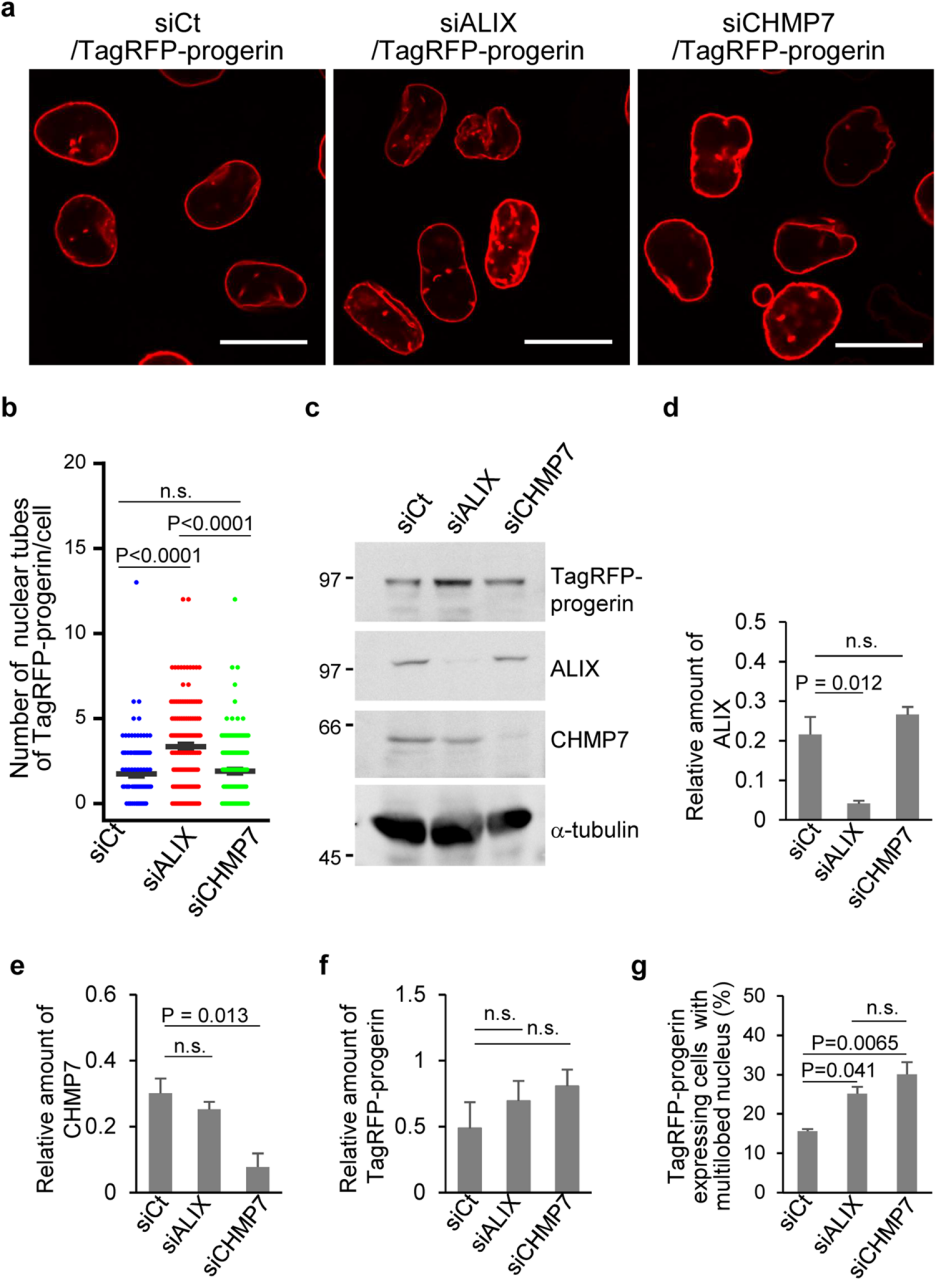
Membrane proliferation induced by the ectopic expression of TagRFP-progerin is de-repressed by ALIX. (**a**) Confocal microscope images of HeLa cells treated with control siRNA (siCt), siRNA to ALIX (siALIX) or siRNA to CHMP7 (siCHMP7) for 48 h and transfected with the TagRFP-progerin expression vector for 24 h. Bars, 20 µm. Images are representative of 3 independent experiments. (**b**) The number of nuclear tube-like structures was measured in 112 (siCt), 160 (siALIX) and 155 (siCHMP7) transfected cells in the experiment presented in panel (**a**). Data are shown as the mean ± SEM. Images are representative of 3 independent experiments. (**c**) Lysates of HeLa cells treated with control siRNA, siRNA to ALIX or siRNA to CHMP7 for 48 h and transfected with the TagRFP-progerin expression vector for 24 h were analyzed by immunoblotting with anti-TagRFP, anti-ALIX, anti-CHMP7 and anti-α-tubulin antibodies. Images are representative of 3 independent experiments. Full-length blots are presented in Supplementary Fig. S3. (**d**–**f**) Quantitation of the amount of ALIX (**d**), CHMP7 (**e**) and TagRFP-progerin (**f**), as indicated, in the immunoblot bands shown in panel (**c**) relative to the amount in the α-tubulin band. (**g**) Percentage of cells (50–160 cells were scored in each experiment) showing a multilobed nucleus in the experiment presented in panel (**a**). Data are shown as the mean ± standard error from 3 independent experiments. Each value is the mean ± standard error of 3 independent experiments. The indicated *p* values were obtained using Tukey’s test. *n.s.* not significant.

**Figure 5:**
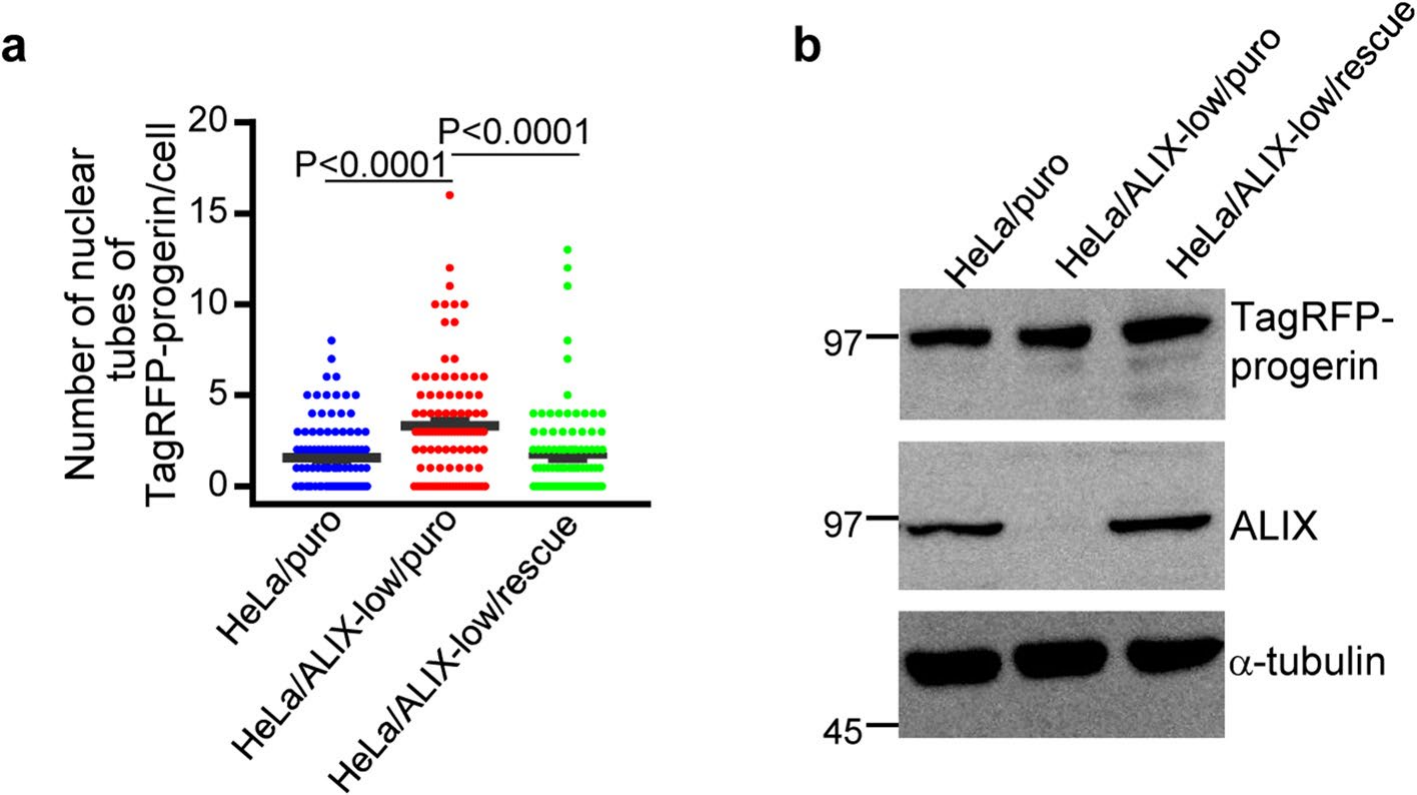
Effects of ectopic expression of ALIX in ALIX-depleted cells. (**a**) HeLa/puro, HeLa/ALIX-low/puro and HeLa/ALIX-low/rescue cells were transfected with the TagRFP-progerin expression vector for 24 h. The number of nuclear tube-like structures was measured in 100 transfected cells. Data are shown as the mean ± SEM and are representative of 3 independent experiments. (**b**) Lysates of HeLa/puro, HeLa/ALIX-low/puro and HeLa/ALIX-low/rescue cells transfected with the TagRFP-progerin expression vector for 24 h were analyzed by immunoblotting with anti-TagRFP, anti-ALIX and anti-α-tubulin antibodies. Images are representative of 3 independent experiments. Full-length blots are presented in Supplementary Fig. S4.

ESCRT-I, ESCRT-II, ALIX and CHMP7 act as adaptors for ESCRT-III that recruit ESCRT-III proteins to their site of action and initiate their assembly for membrane remodeling^25^. Among them, ALIX and CHMP7 participate in the nuclear functions of ESCRT-III complexes^2,7,8^. We next examined effects of ALIX or CHMP7 on the progerin-induced NM deformation by knocking down each of the adaptors with siRNA. Concordant with the effects of CHMP4B knockdown, ALIX depletion significantly increased the number of TagRFP-progerin tube-like structures (Fig. 4a–d). The effect of ALIX depletion in these cells was significantly rescued by the ectopic expression of ALIX (Fig. 5a,b). In contrast, CHMP7 depletion had no obvious effect on TagRFP-progerin tube-like structures (Fig. 4a–c,e), and depletion of ALIX or CHMP7 had little effect on accumulation of TagRFP-progerin in the cells (Fig. 4f). We note that ALIX or CHMP7 depletion can induce the formation of multilobular nuclei^3,21^. In agreement with these previous reports, treatment with ALIX or CHMP4B siRNAs significantly augmented the frequencies of cells that contained a multilobular nucleus (Fig. 4g). These results indicate that ALIX, but not CHMP7, is required to suppress progerin-induced NM deformation.

### Rapamycin treatment down-regulates progerin-induced NM deformation in a manner dependent on ESCRT-III

**Figure 6:**
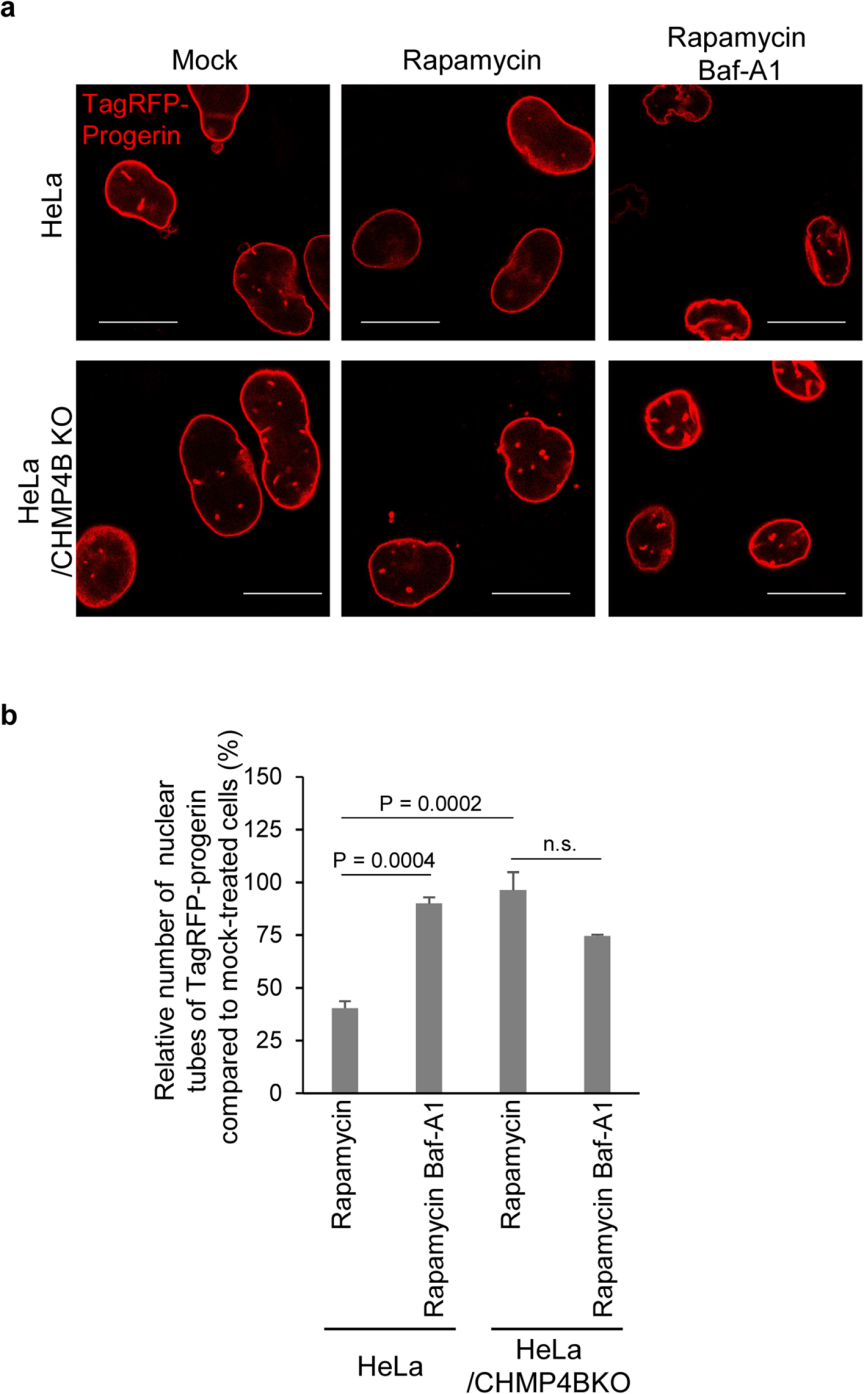
Effect of rapamycin treatment in ESCRT-III depleted cells. (**a**) Confocal microscope images of HeLa cells and HeLa/CHMP4BKO cells were transfected with the plasmid expressing TagRFP-progerin for 24 h followed by mock or rapamycin treatment in the presence or absence of Bafilomycin A1 (Baf-A1) for 24 h. Bars, 20 µm. Images are representative of 3 independent experiments. (**b**) Relative number of trans-nuclear tubes of HeLa cells and HeLa/CHMP4BKO cells transfected with the plasmid expressing TagRFP-progerin, followed by the rapamycin treatment in the presence or absence of Baf-A1 to these of mock treated cells as described in (**a**). Data are shown as the mean ± SEM of 3 independent experiments. The indicated *p* values were obtained using Tukey’s test. *n.s.* not significant.

To investigate whether ESCRT-III co-operates with autophagy in order to suppress progerin-induced NM deformation, we used the chemical tools rapamycin and bafilomycin A1 (Baf-A1) to activate or inhibit autophagy, respectively, by affecting lysosome-based degradation. In agreement with earlier reports^17^, rapamycin treatment significantly decreased the number of TagRFP-progerin tube-like structures in HeLa cells, whereas concomitant treatment with Baf-A1 antagonized this effect. These results verified an earlier study^17^ that showed autophagy is required for rapamycin-dependent suppression of progerin-induced NM deformation. In contrast, rapamycin treatment had little effect on the number of TagRFP-progerin tube-like structures in HeLa/CHMP4BKO cells, and additional treatment with Baf-A1 did not increase the number of TagRFP-progerin tube-like structures (Fig. 6a,b). These results indicate that ESCRT-III is required for suppression of progerin-induced NM deformation by rapamycin and that autophagy and ESCRT-III act in suppression of progerin-induced NM deformation by rapamycin in the same pathway. Alternatively, upon reduction of progerin levels, rapamycin-treated cells might initiate ESCRT-III-dependent membrane repair. In any case, these series of observations suggest that autophagy suppresses progerin-induced NM deformation in a manner dependent on ESCRT-III.

### ESCRT-III down-regulates INM deformation in HGPS fibroblasts

**Figure 7:**
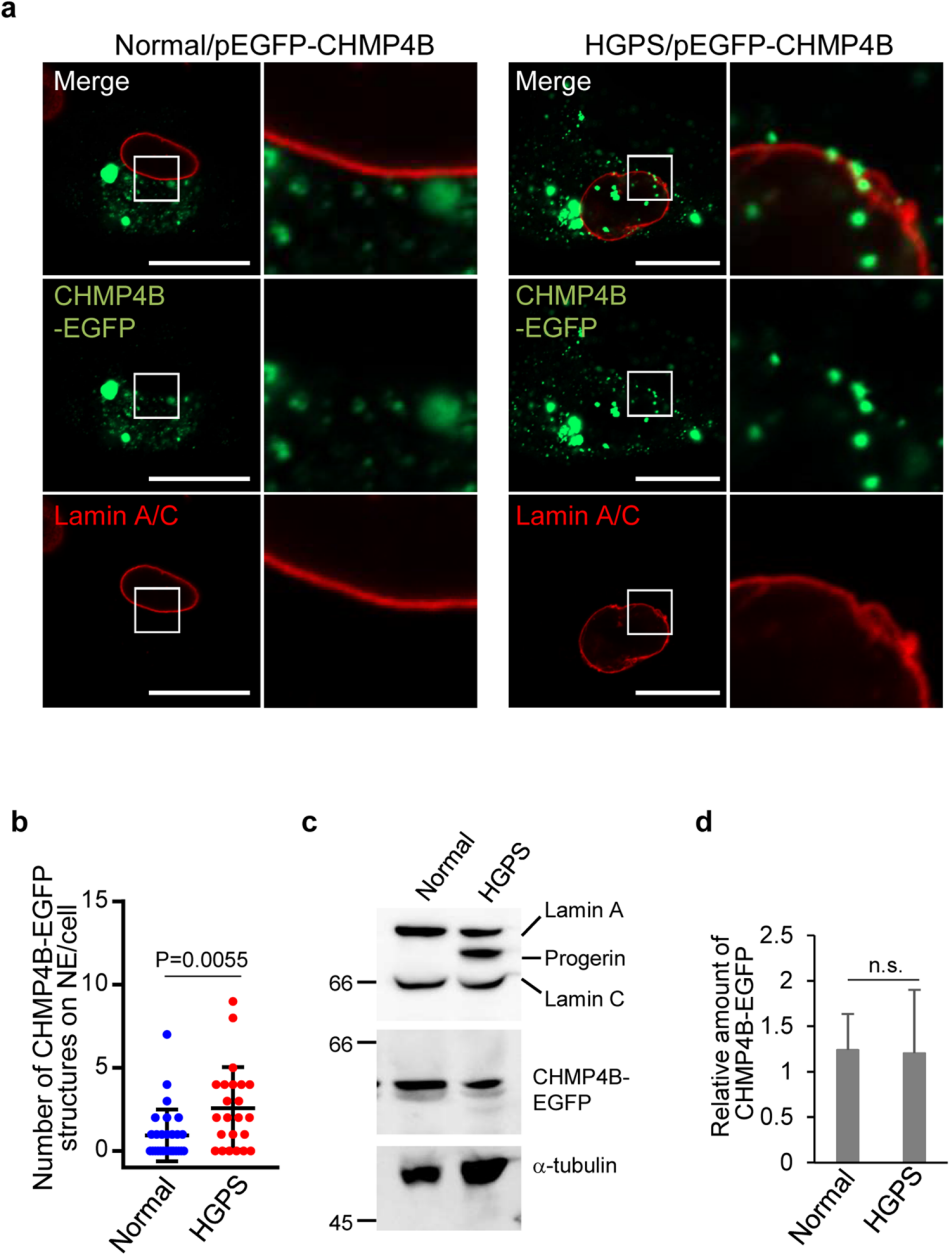
ESCRT-III is recruited to the sites of membrane proliferation in fibroblasts from an HGPS patient. (**a**) Confocal microscope images of normal (AG03512) or HGPS fibroblasts (AG11513) transfected with the pEGFP-CHMP4B for 48 h were stained with anti-lamin A/C antibody. The right panels are the magnified image of the boxed area in the left panels. Bars, 20 µm. Images are representative of 3 independent experiments. (**b**) The number of CHMP4B dots on the NM in the experiment in (**a**) was measured for 29 (normal) or 23 (HGPS) transfected cells. Data are shown as the mean ± SEM and are representative of 3 independent experiments. (**c**) Lysates of normal or HGPS cells transfected with the pEGFP-CHMP4B for 48 h were analyzed by immunoblotting with anti-lamin A/C, anti-EGFP and anti-α-tubulin antibodies. Images are representative of 3 independent experiments. Full-length blots are presented in Supplementary Fig. S5. (**d**) Quantitation of the amount of CHMP4B-EGFP, as indicated, in the immunoblot bands shown in panel (**c**) relative to the amount in the α-tubulin band. Each value is the mean ± standard error of 3 independent experiments. *n.s*. not significant (by the unpaired Student’s *t* test).

To conclude the study, we investigated the effect of ESCRT-III in HGPS fibroblasts. As observed with HeLa cells ectopically expressing TagRFP-progerin, tube-like structures detected by lamin A/C antibody using confocal microscopy were consistently detectable in HGPS fibroblasts wherein CHMP4B-EGFP was ectopically expressed, whereas these structures were barely detectable in fibroblasts from a healthy donor (normal fibroblasts) (Fig. 7a). The number of punctate structures of CHMP4B-EGFP detected at the NM in HGPS fibroblasts was significantly higher than in normal fibroblasts (Fig. 7b), although the total CHMP4B-EGFP levels were similar in these fibroblasts (Fig. 7c,d). These results indicate that CHMP4B is preferentially recruited to the NM in HGPS fibroblasts. However, we cannot exclude the possibility that the level of exogenous CHMP4B-EGFP is higher than that of endogenous CHMP4B, which could affect the physiological function of ESCRT-III in these experiments.

**Figure 8:**
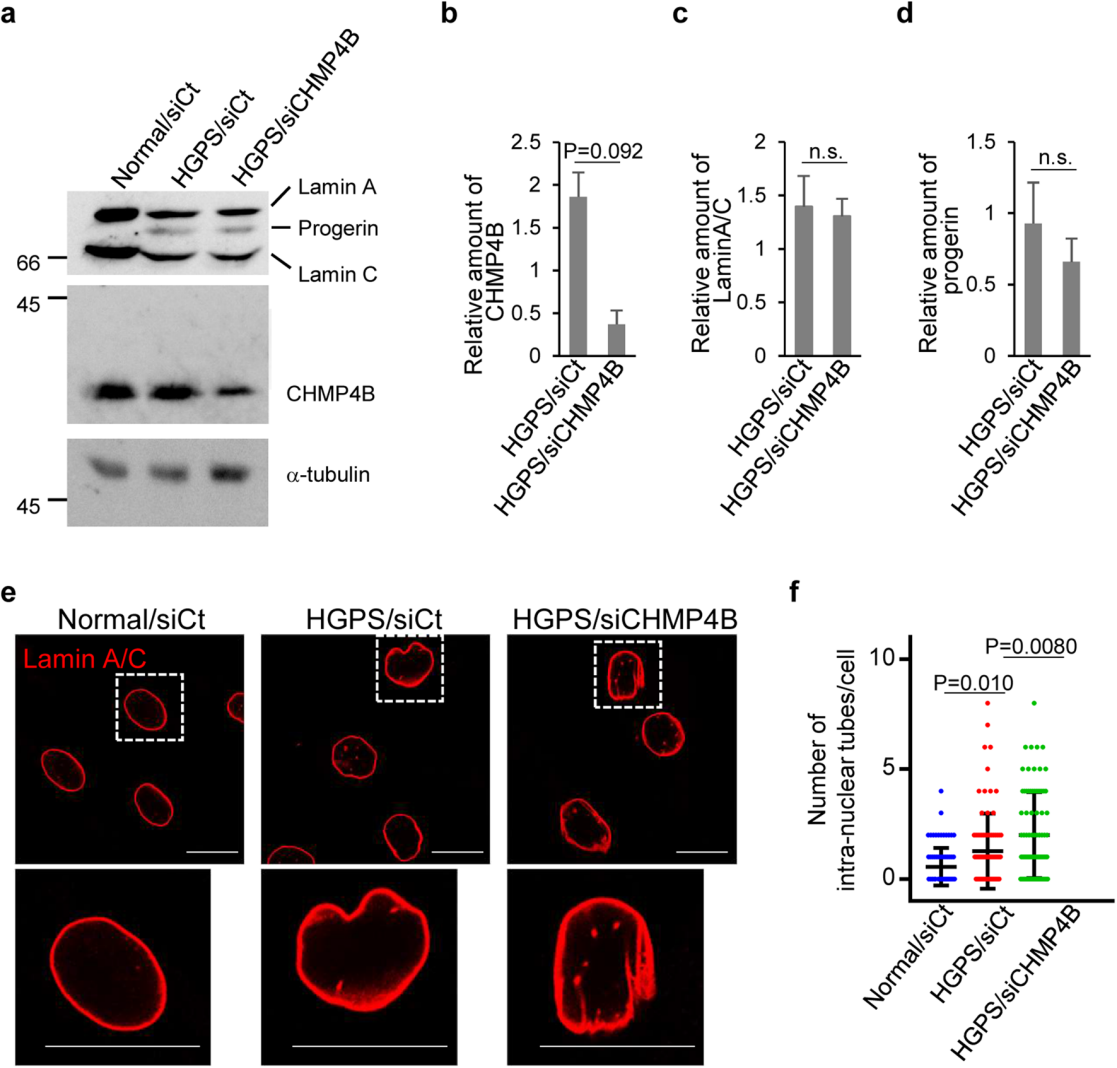
Roles of ESCRT-III in fibroblasts from an HGPS patient. (**a**) Lysates of normal or HGPS cells treated with control siRNA or siRNA to CHMP4B for 48 h were analyzed by immunoblotting with anti-lamin A/C, anti-CHMP4B and anti-α-tubulin antibodies. Images are representative of 3 independent experiments. Full-length blots are presented in Supplementary Fig. S6. (**b**–**d**) Quantitation of the amount of CHMP4B (**b**), lamin A/C (**c**) or progerin (**d**) in the immunoblot bands shown in panel (**a**) relative to the amount in the α-tubulin band. Each value is the mean ± standard error of 4 independent experiments. The indicated *p *values were obtained using an unpaired Student’s *t* test. *n.s.* not significant. (**e**) Confocal microscope images of HGPS cells treated with the indicated siRNA for 48 h and stained with anti-lamin A/C antibody. The lower panels are the magnified image of the boxed area in the upper panels. Bars, 20 µm. Images are representative of 3 independent experiments. (**f**) The number of intra-nuclear tubes in 85 HGPS cells in the experiment in (**e**) was measured. Data are shown as the mean ± SEM and are representative of 3 independent experiments. The indicated *p* values were obtained using Tukey’s test.

**Figure 9:**
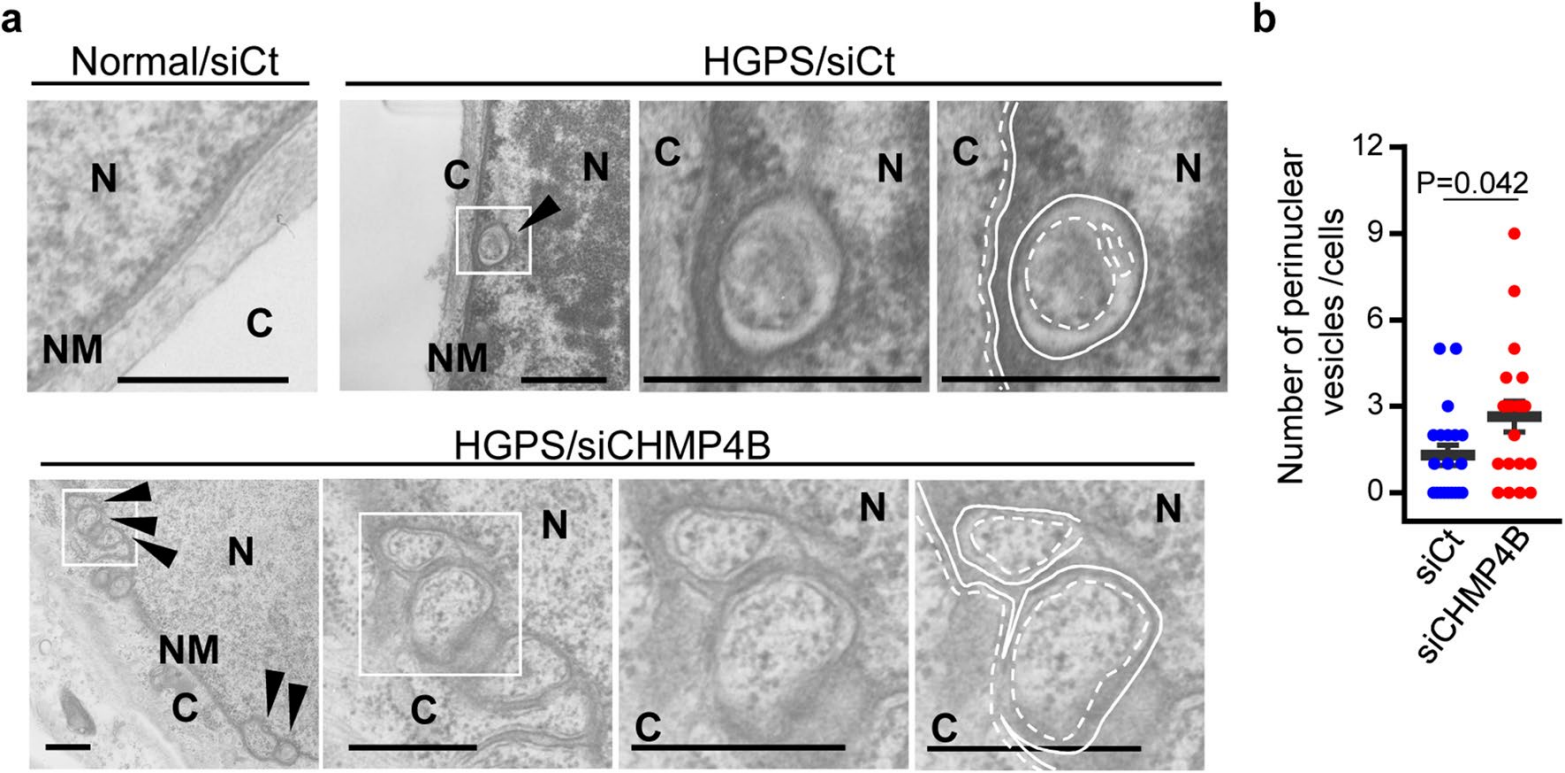
Roles of ESCRT-III in fibroblasts from an HGPS patient. (**a**) Electron microscope images of normal (AG03512) or HGPS (AG11513) human skin fibroblasts, treated with the indicated siRNA for 48 h. Arrowheads indicate vesicles in the perinuclear spaces. The right panel is the magnified image of the boxed area in the left panel. Straight lines indicate the INM and dotted lines indicate the ONM and the membrane of vesicles in the perinuclear spaces. N, nucleus; C, cytoplasm; NM, nuclear membrane. Bars, 500 nm. Images are representative of 3 independent experiments. (**b**) The perinuclear vesicle structures in siRNA treated HGPS cells in the experiment in (**a**) were quantitated. Data are shown as the mean ± SEM for 20 cells and are representative of 3 independent experiments.

We then investigated the effect of CHMP4B depletion on NM deformation in HGPS fibroblasts by knocking down CHMP4B with siRNA. CHMP4B depletion (Fig. 8a–d) significantly increased the number of tube-like structures of lamin A/C in HGPS fibroblasts (Fig. 8e,f) without affecting accumulation of lamin A/C and progerin (Fig. 8c,d). These results indicate that ESCRT-III is required to suppress NM deformation induced in HGPS fibroblasts. Notably, at the ultrastructural level, membranous INM invaginations containing vesicle-like structures, similar to those observed during vesicle-mediated nucleocytoplasmic transport of RNPs in *Drosophila* cells^26^, were easily detectable in HGPS fibroblasts, and the number of invaginated structures was significantly increased by CHMP4B depletion (Fig. 9a,b). This is again consistent with the effects of depleting the CHMP4 homolog, shrub, in *Drosophila* cells^8^. In normal fibroblasts, these structures were not observed (Fig. 9a).

## Discussion

We recently reported that ESCRT-III mediates scission at the INM during vesicle-mediated nucleocytoplasmic transport^8^, which is a unique mechanism for the nuclear export of macromolecular complexes^27^. In this system, a macromolecular complex in the nucleus buds through the INM to form a vesicle in the perinuclear space that then fuses with the outer nuclear membrane (ONM) to release the complex into the cytoplasm^28^. We also reported that ESCRT-III is required for the proper integrity of the INM in human cells. Thus, CHMP4B depletion in HeLa induced tube-like structures and INM proliferation^8^, and these effects were phenocopied in the present study in HeLa cells ectopically expressing progerin. Notably, mutations in genes encoding INM-associated proteins such as laminA/C and Emerin, which are associated with various hereditary diseases such as HGPS, lead to aberrant accumulation of mutant proteins such as progerin, thereby inducing aberrant nuclear morphologies that include a multi-layered INM^10,29^. Furthermore, overexpression of various INM-associated proteins was reported to cause aberrant NM proliferation^30–32^. In light of all these data, we suggested a model in which ESCRT-III ensures INM integrity by regulating vesicle-mediated nucleocytoplasmic transport of excess INM and INM proteins in normal cells^8^. In our present study, we extended our previous observations by investigating the role of ESCRT-III in progerin-induced NM deformation and showed that ESCRT-III was required for suppression of progerin-induced INM proliferation. We also demonstrated that autophagy suppresses progerin-induced NM deformation in a manner dependent on ESCRT-III machinery. Notably, INM invaginations containing vesicle-like structures, which are the hallmarks of vesicle-mediated nucleocytoplasmic transport as observed in herpesvirus-infected cells and *Drosophila* cells^8,26,28,33–36^, were consistently detectable in HGPS fibroblasts. Furthermore, the number of these structures was increased by depletion of CHMP4B, an essential regulator of vesicle-mediated nucleocytoplasmic transport. These observations led to the hypothesis, which is in agreement with the model suggested in our earlier report^8^, ESCRT-III suppresses progerin-induced INM deformation by regulating vesicle-mediated nucleocytoplasmic transport of deformed INM and probably progerin as well for progerin degradation by autophagy in the cytoplasm. It seems likely that in normal cells, ESCRT-III is able to maintain proper integrity of the INM in normal cells, but that in HGPS fibroblasts progerin saturates the system and therefore induces INM deformation.

In addition to controlling scission of the INM during vesicle-mediated nucleocytoplasmic transport and maintaining INM integrity, ESCRT-III complexes have other nuclear functions. These include post-mitotic resealing of the NM and repair of NM ruptures produced during migration of cancer and immune cells through tight interstitial spaces^2,3,6,7^, both of which require CHMP7 as an adaptor for ESCRT-III. In this study, we showed that ALIX, but not CHMP7, is required for suppression of progerin-induced INM deformation. These results eliminate the possibility that the effects of ESCRT-III depletion were caused due to defects in post-mitotic resealing of NM or repair of NM rupture. Notably, the role of ESCRT-III in vesicle-mediated nucleocytoplasmic is dependent on ALIX^8^. Together, these observations further support our hypothesis described above.

HGPS patients typically die at a mean age of 14.6 years^9^ and a therapeutic approach for HGPS has not yet been established^9^. In this study, we showed that the ALIX-mediated ESCRT-III pathway inhibits the major progerin-induced cellular phenotype. Therefore, modulation of the ALIX-ESCRT-III pathway could be a potential therapeutic strategy for HGPS and may co-operate with drugs such as rapamycin and MG132 that induce autophagy. It has been reported that herpesviruses substantially activate the ALIX-mediated ESCRT-III pathway for nuclear export of their nascent nucleocapsids without affecting the INM integrity^8^. Therefore, mimicking this viral activity would be a potential strategy to improve HGPS cellular phenotype.

## Methods

### Cells

HeLa, HeLa/puro, HeLa/CHMP4B-EGFP, HeLa/CHMP4BKO, HeLa/CHMP4BKO/puro, HeLa/CHMP4BKO/CHMP4B-EGFP, HeLa/ALIX-low, HeLa/ALIX-low/puro, HeLa/ALIX-low/rescue cells were described previously^8,37^. Normal (AG03512) and HGPS (AG11513) human skin fibroblasts obtained from the National Institute of Aging (NIA) Aged Cell Repository (distributed by the Coriell Institute, Camden, NJ) were maintained in DMEM containing 15% fetal bovine serum (FBS). In cases using these fibroblasts, experiments were performed within 18 passages from their establishment.

### Plasmids

pTagRFP-N3 was constructed by substituting the EGFP coding region in pEGFP-N3 with a DNA fragment containing the TagRFP coding region, amplified by PCR from pTagRFP-C (Evrogen, Moscow, Russia). A plasmid encoding a fusion protein of TagRFP and the lamin A coding region (pTagRFP-lamin A) was constructed by cloning lamin A cDNA, amplified by PCR from HeLa cell cDNA, into pcDNA3.1(–), followed by cloning TagRFP into the lamin A N-terminus. Similarly, a plasmid was constructed encoding a fusion protein of Tag-RFP and progerin (i.e. HPGS-associated mutant lamin A) (pTagRFP-progerin), with a 150-bp deletion near the lamin A C-terminus (nucleotides 1819–1968).

### Antibodies

These studies used commercial mouse monoclonal antibodies against ALIX (sc-53540; Santa Cruz Biotechnology, Dallas, TX), α-tubulin (DM1A; Sigma, St. Louis, MO), lamin A/C (sc-7292; Santa Cruz Biotechnology); commercial rabbit polyclonal antibody against GFP (598; MBL, Nagoya, Japan), TagRFP (AB233; Evrogen, Moscow, Russia), CHMP4B (ab105767; Abcam, Cambridge, UK).

### Knockdown experiments

Small interfering RNAs (siRNAs) with target sequences to ALIX (5′-GAACAAAUGCAGUGAUAUA-3′), CHMP7 (5′-AGGUCUCUCCAGUCAAUGA-3′ and 5′-GCAATAGGCATTTTACCAA-3′), CHMP4B (5′-CGAUAAAGUUGAUGAGUUA-3′) and a control sequence were purchased from Dharmacon (Lafayette, CO). HeLa cells were treated with 1 nM siRNA for 48 h and then transfected with the TagRFP-progerin expression vector for further analysis. Normal or HGPS fibroblasts were treated with 1 nM siRNA and analyzed after 48 h.

### Immunoblotting, immunofluorescence and live-cell imaging

Immunoblotting was performed as described previously^8^. For immunofluorescence, the cells were fixed and stained with the indicated antibodies as described previously^38^. For live-cell imaging, cells were transfected with the indicated expression vectors and images acquired by the LSM800 microscope (Zeiss, Oberkochen, Germany) were analyzed with the colocalization function in ZEN2.1 software (Zeiss) as previously reported^8,39^.

### Electron microscopy

For these studies, either (1) HeLa or HeLa/CHMP4BKO cells transfected with the TagRFP-progerin expression plasmid for 24 h or (2) normal or HGPS fibroblasts treated with control siRNA or siRNA to CHMP4B for 48 h were then embedded, sectioned, stained and examined by ultrathin-section electron microscopy as described previously^8,40^. Immunoelectron microscopy was performed as described previously^8,41,42^. Briefly, HeLa-CHMP4B-EGFP cells transfected with the TagRFP-progerin expression plasmids for 24 h were fixed with 2% paraformaldehyde and 1% glutaraldehyde on ice for 2 h, post-fixed with 2% osmium tetroxide on ice for 2 h, washed with distilled water, dehydrated with an ethanol gradient series, incubated in propylene oxide, and embedded in an Epon 812 resin mixture. Ultrathin sections were prepared on grids as described previously^8,40^. After a phosphate buffered saline (PBS) wash, the sections were incubated with 10% human serum and 5% bovine serum albumin in PBS and then with anti-GFP rabbit polyclonal antibody. The sections were then washed with PBS and incubated with goat anti-mouse IgG conjugated to 10 nm gold particles. After immunostaining, the sections were stained with 2% uranyl acetate and Reynold’s lead citrate and examined by transmission electron microscopy. For statistical analyses, the length of NM was calculated by ImageJ software (NIH, Bethesda, MD).

### Rapamycin treatment

HeLa or HeLa/CHMP4BKO cells were transfected with the plasmid expressing TagRFP-progerin for 24 h. Then, these cells were mock-treated or treated with 680 nM of rapamycin in the presence or absence of 200 nM of bafilomycin A1 (Baf-A1) and analyzed after another 24 h.

### Statistical analysis

For the comparison of two groups, statistical analysis was performed using the unpaired Student’s *t* test. Tukey’s test was used for multiple comparisons. A *p* value > 0.05 was considered not significant (n.s.).

## Supplementary information

Supplementary Information.

## Data Availability

The authors declare that the data supporting the findings of this study are available within the article, or are available on request.
