# Supplementary material for: ESCRT-III controls nuclear envelope deformation induced by progerin
Source: Sci Rep. 2020 Nov 2;10:18877. doi: 10.1038/s41598-020-75852-6 (PMC7606583; doi:10.1038/s41598-020-75852-6)
Supplement: Supplementary file 1 — Supplementary Information. [file 41598_2020_75852_MOESM1_ESM.pdf]

Supplementary information

ESCRT-III controls nuclear envelope deformation induced by progerin

Jun Arii[a, b, c, d], Fumio Maeda[a, b], Yuhei Maruzuru[a, b], Naoto Koyanagi[a, b, c], Akihisa Kato[a, b, c], Yasuko Mori[d] and Yasushi Kawaguchi[a, b, c]*

[a]Division of Molecular Virology, Department of Microbiology and Immunology, The Institute of Medical Science, The University of Tokyo, Tokyo, Japan
[b]Department of Infectious Disease Control, International Research Center for Infectious Diseases, The Institute of Medical Science, The University of Tokyo, Tokyo, Japan
[c]Research Center for Asian Infectious Diseases, The Institute of Medical Science, The University of Tokyo, Tokyo, Japan
[d]Division of Clinical Virology, Center for Infectious Diseases, Kobe University Graduate School of Medicine, Kobe, Hyogo, Japan.

*Address correspondence to:
Dr. Yasushi Kawaguchi
Division of Molecular Virology
Department of Microbiology and Immunology
The Institute of Medical Science
The University of Tokyo
4-6-1 Shirokanedai, Minato-ku, Tokyo 108-8639, Japan.
Phone: 81-3-6409-2070
Fax: 81-3-6409-2072
E-mail: ykawagu@ims.u-tokyo.ac.jp

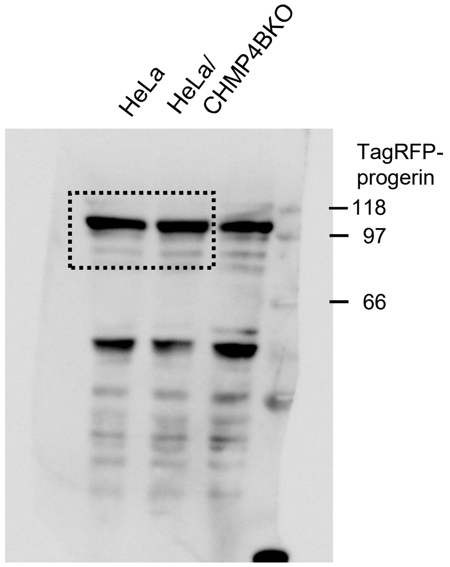

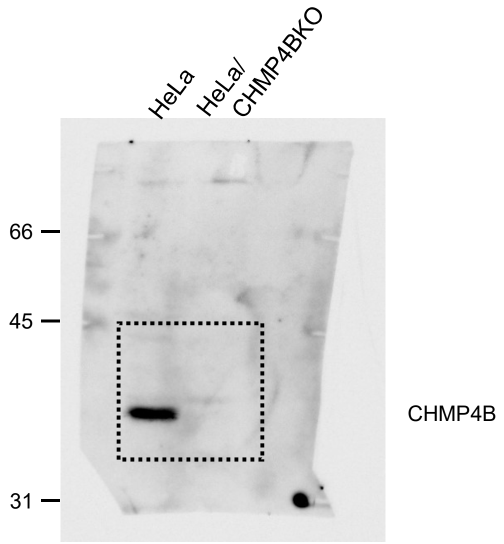

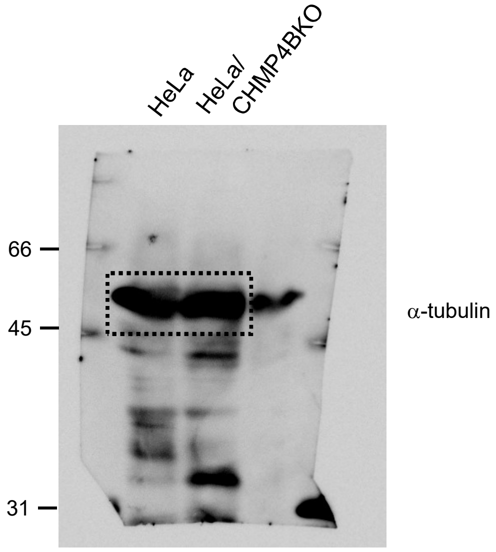

J. Arii et al., Figure S1 (original data of Figure. 2d)

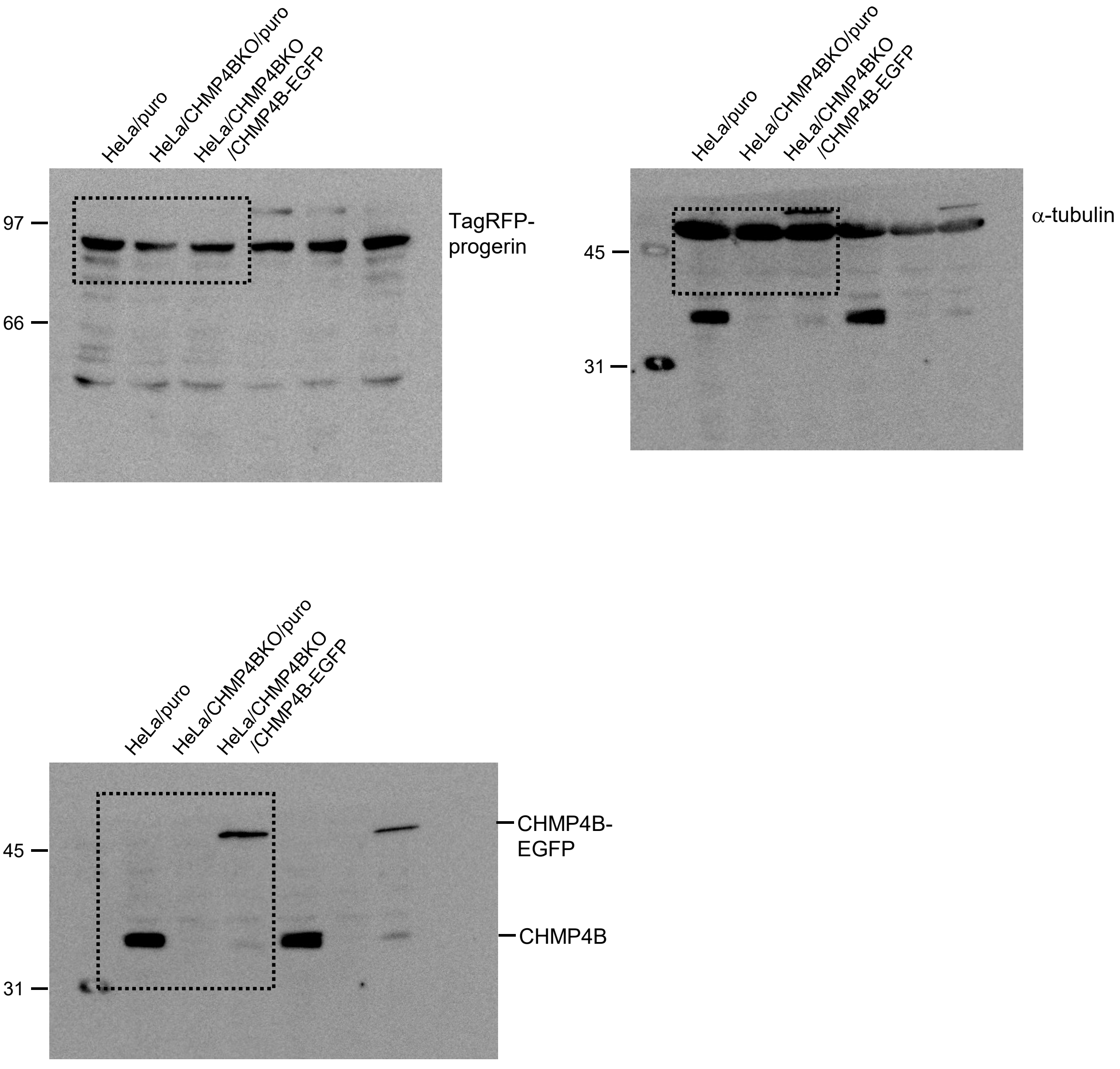

J. Arii et al., Figure S2 (original data of Figure. 3b)

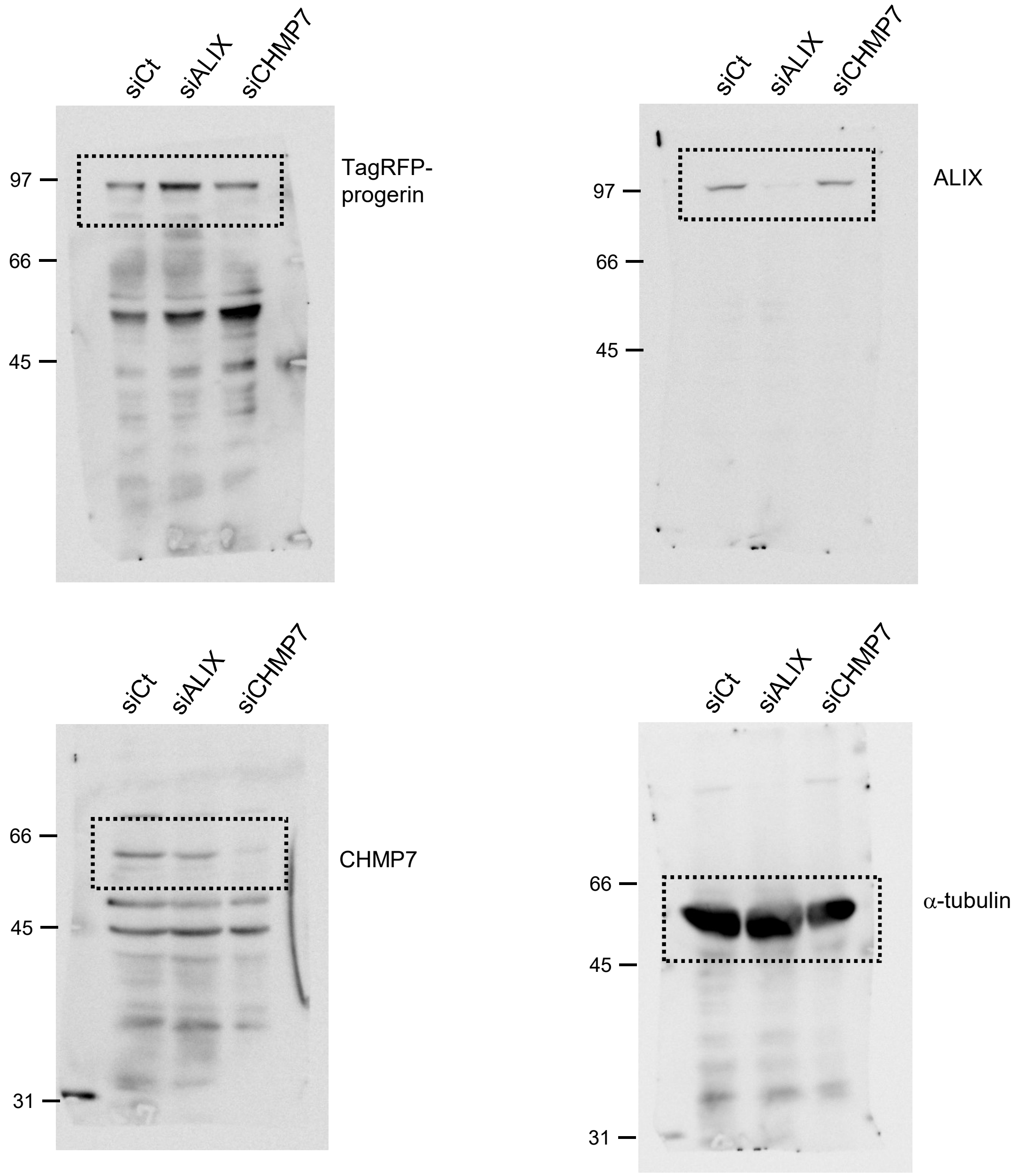

J. Arii et al., Figure S3 (original data of Figure. 4c)

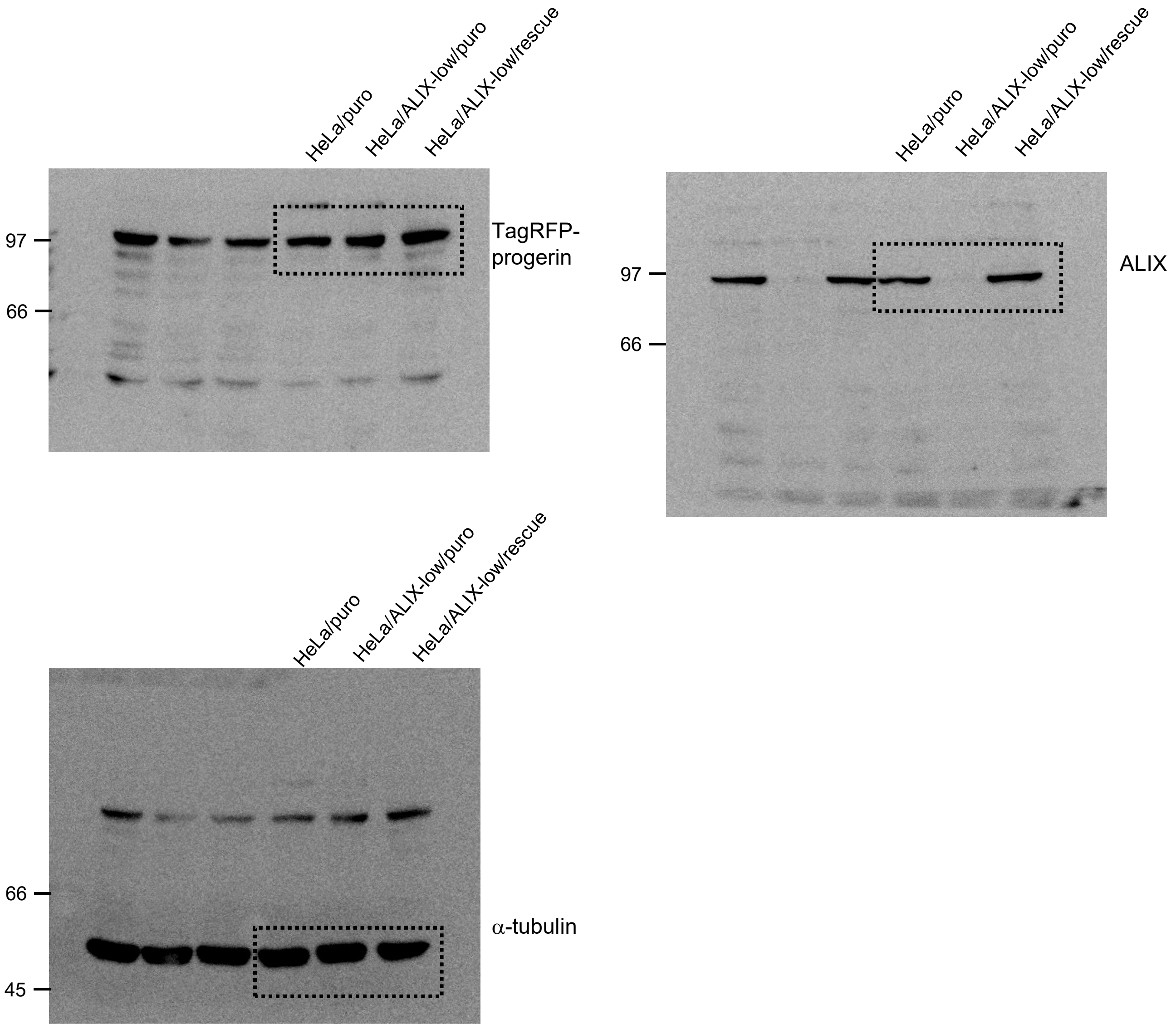

J. Arii et al., Figure S4 (original data of Figure. 5b)

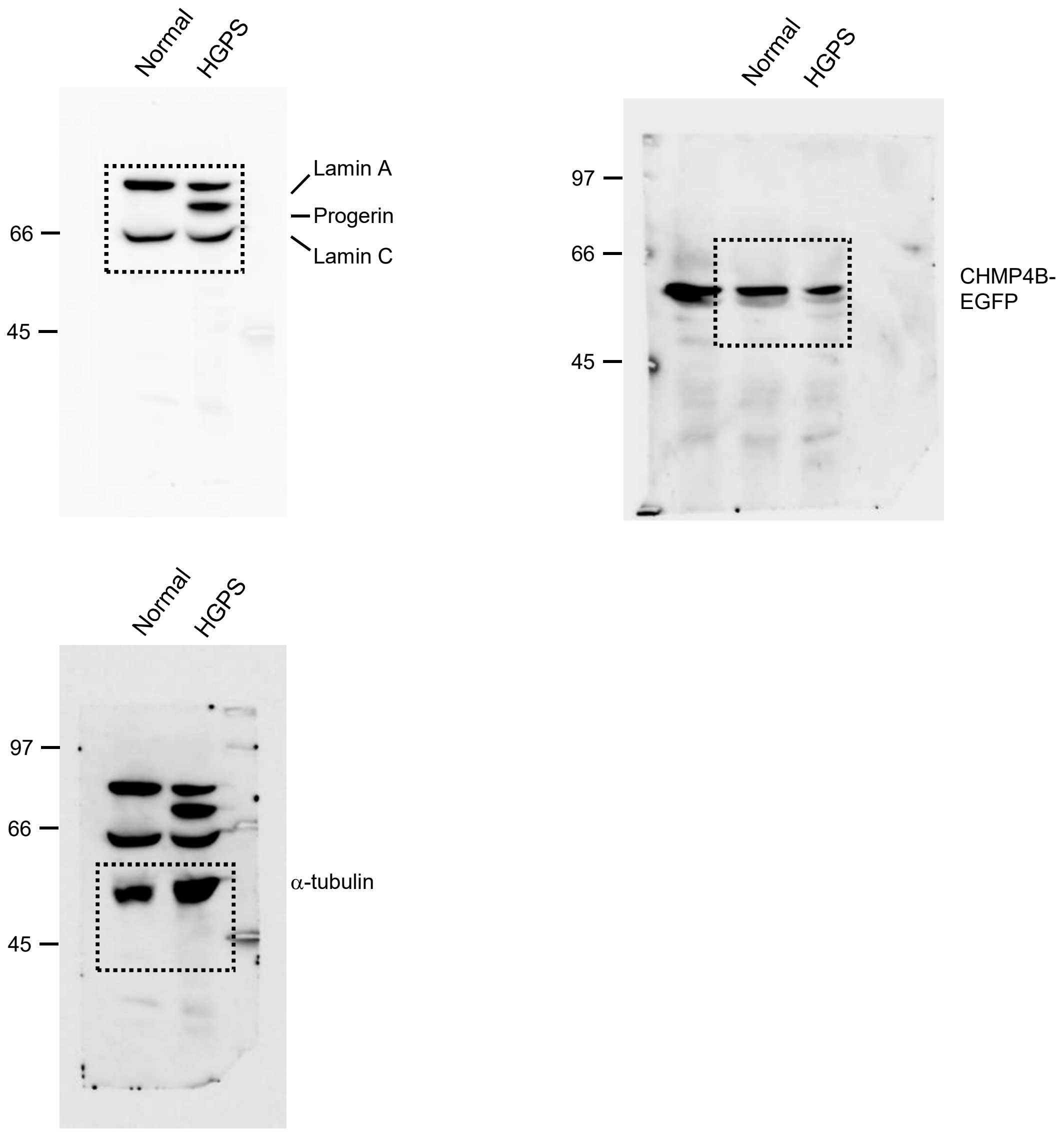

J. Arii et al., Figure S5 (original data of Figure. 7c)

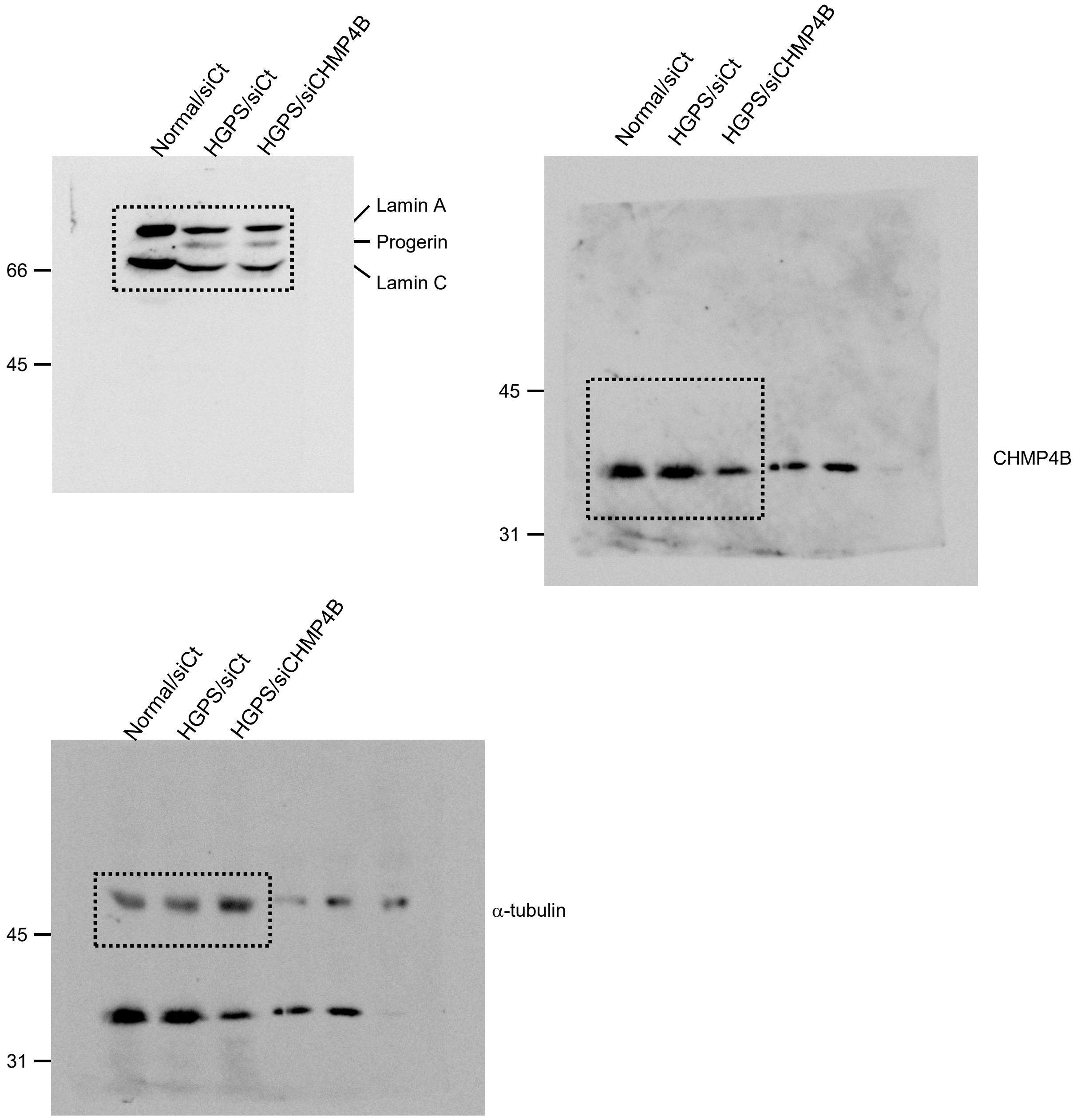

J. Arii et al., Figure S6 (original data of Figure. 8a)
